# The I.C.A.A. as a Clearing House

**Published:** 1917-07-07

**Authors:** 


### THE FEDERATION OF HOSPITAL TREATMENT FOR CHILDREN.

### The I.C.A.A. as a Clearing House.

IN an article recently contributed to *Maternity and Child Welfare*,* Dr. C. J. Macalister discusses certain defects in the present system of caring for children's diseases in Liverpool, and proposes as a remedy the further extension of certain functions which are carried out there by the Invalid Children's Aid Association. Dr. Macalister's argument is based on the assertion—and there are perhaps few members of the medical profession in Liverpool better qualified to make it—that the in-patient accommodation for children in the Liverpool general and special hospitals is insufficient to yield the curative results necessary to produce the healthiest possible next adult generation. He says that the out-patient clinics are hampered because there are not sufficient cots for all the cases which in their own best interests should receive in-patient treatment. The result is that many cases are retained as out-patients, or are put on waiting-lists, and fail to be restored to health as speedily and completely as would be the case if more in-patient accommodation were available for them.

In order to obviate the prejudice to public interests which this condition of things clearly indicates in Dr. Macalister's opinion, he proposes a system by which the available beds shall be utilised in the most profitable and economical manner. He does not suggest the other alternative—that is, the erection of new clinics for in-patients. But he notes that extensions of the capacity of existing institutions are going on in more than one direction. What he wishes to promote is the co-ordination of a considerable number of therapeutic agencies already at work independently, and particularly of the various convalescent homes which undertake the after-care of cases from the Liverpool children's clinics. The machinery for such co-ordination already exists, declared Dr. Macalister, and the pivot around which federation can be arranged is the Invalid Children's Aid Association.

As an example of the "clearing house" functions which he would like to see allotted to the Association, he mentions the arrangements which have been made between it and the Royal Southern Hospital, Liverpool, where a large number of sick children of all types has to be dealt with. An officer of the Association, chosen for experience and ability as a social worker, attends the out-patient clinic of the Royal Southern Hospital, and the medical officer in charge, whenever he encounters a case requiring anything more than out-patient treatment, indicates to the Invalid Children's Aid Association worker the special kind of institution for which the case is best suited. Acute cases are, of course, admitted in the ordinary way to the wards of the hospital. Infants are sent either to the Liverpool Country Hospital for Children at Heswall, which is now seeking to extend its accommodation from the 150 beds at present open, and may in time grow to the 500 for which it was originally planned, or to the Leasowe Hospital, where fifty beds have recently been opened for wasting infants and similar cases. Other cases are sent to the "Ellen Gonner" Home at Hoylake, to the Children's Convalescent Home at West Kirby, to St. Anne's-on-Sea, to Southport, or to any other place where beds may be at the moment available.

If this system can be worked by one hospital, Dr. Macalister argues, why should it not be carried out at all of them? It would be quite a simple matter, he thinks, "to have an Invalid Children's Aid Association officer in attendance at every children's clinic, and so to secure the systematic treatment of the bulk of the children seeking help in the city." When the Heswall expansion to which he looks forward is brought about, there will be a considerable relief to the beds in the city hospitals, many of which will then be available for the reception of cases of the acuter types, which at present have often to be refused or deferred.

There is a further, if indirect, public advantage which Dr. Macalister foresees from the adoption of his suggestions. As a branch of this federation, or "clearing house," work, he looks forward to the establishment of schemes of education for social workers and for women, who undertake the duties of visitors under the Invalid Children's Aid Association and its federal institutions. Their education, he believes, will lead to the teaching of the public concerning the general hygienic management of children. As a consequence of such education of the parents, rickets and other metabolic diseases might be materially diminished in number; thus, of course, lessening the strain on the institutions by cutting off at the source part of the supply of cases with which they have to deal. Prevention is better than cure, certainly, and nowhere more so than in the conservation of infant life.

* May 1917, p. 181.

